# The Type of Substance Use Mediates the Difference in the Odds of Sexual Risk Behaviors Among Adolescents of the United States

**DOI:** 10.7759/cureus.17264

**Published:** 2021-08-17

**Authors:** Saral Desai, Nishat Kulkarni, Sanila Rehmatullah

**Affiliations:** 1 Department of Psychiatry, Brookdale University Hospital Medical Center, Brooklyn, USA

**Keywords:** risky sexual behaviors, substance abuse, substance use, child and adolescent psychiatry, anabolic steroids, alcohol, marijuana, cocaine, sexually transmitted diseases, sexual risk behavior

## Abstract

Background

Although the relationship between sexual risk behaviors and substance use has been established, It remains to be studied if different types of substances have differences in the odds of sexual risk behaviors. Therefore, we aimed to identify the prevalence of sexual risk behaviors in high school students of the United States (US) and study the difference in the odds of sexual risk behaviors for various substances.

Methods

We performed a retrospective cross-sectional study using Youth Risk Behavior Surveillance System (YRBSS) data of 2019 that nationally represents US high school students in grades 9-12. We identified individuals with sexual risk behaviors as participants with four or more lifetime sexual partners and who did not use a condom during the last intercourse.

Results

Out of 11,191 participants, 463 (3.9%) engaged in sexual risk behaviors. The prevalence of substance use, including anabolic steroids (11.5 vs. 1.1%), cocaine (27.2 vs. 2.0%), marijuana (87.1 vs. 34.7%), alcohol (92.4 vs. 54.3%), e-cigarette (90.3 vs. 48.0%), and traditional cigarette (62.2 vs. 21.6%) was higher in participants with sexual risk behaviors compared to participants with no sexual risk behaviors (p<0.0001 for all substances). In regression analysis, anabolic steroid use was associated with the highest odds of sexual risk behaviors (adjusted odds ratio (aOR):4.87, 95%CI: 2.48-9.57; p<0.0001) followed by cocaine (aOR:3.80, 1.80-8.00; p=0.001), marijuana (aOR:3.36, 1.64-6.89; p<0.0001), alcohol (aOR:2.41, 1.05-5.55; p=0.039), electronic vapor products (2.05, 1.004-4.19; p=0.049), and traditional cigarette use (aOR:1.58, 1.10-2.28; p=0.016). We did not find a statistically significant increase in the odds of sexual risk behaviors for the rest of the substances.

Conclusion

Although the prevalence of sexual risk behaviors is low, the prevalence of substance use is significantly higher in participants with sexual risk behaviors. Among the different types of substances, anabolic steroid use has the highest odds of sexual risk behaviors. Therefore, clinicians should remain vigilant for anabolic steroid use when screening adolescents for substance use. Further large-scale randomized studies are needed to study the effects of anabolic steroids on sexual risk behaviors.

## Introduction

According to the CDC, one in five people in the United States (US) have had a sexually transmitted disease (STD) and has an annual cost of nearly 16 billion dollars [1]. STDs like chlamydia, gonorrhea, and syphilis combined accounted for $1.1 billion in direct medical expenses, and young people accounted for about 60% of those costs [1]. America's youth also shoulder a large portion of the annual burden of STDs. In 2018, 45.5% of all new STDs occurred among young people [1]. Growth in the rates of STDs and associated infection costs has created a sense of urgency in understanding the individual and situational factors that increase one's risk for disease. One such factor, substance use before sexual activity, has been shown to increase the likelihood of unsafe sexual intercourse [2]. Previous studies have shown that adolescents with substance use are more likely to become sexually active at an earlier age, have more sexual partners, and are more likely to have unprotected sex [3-5]. According to the Surgeon General's Report in 2016, substance use, including alcohol and other illicit drugs, is a growing problem in the US [6]. Thus, it is critical to identify if all substances or just specific substances are associated with sexual risk behaviors. The precise definition of risky sex or high-risk sexual behaviors has varied throughout the literature. Different studies have explored different sexual risk behaviors and studied their association with various substances [7].

One study found that increasing intensity of alcohol use is associated with an increased number of sexual partners; however, alcohol did not affect chances of unprotected intercourse [8]. Another study found that alcohol with co-ingestion of marijuana increases sexual risk behaviors while marijuana use alone does not [9]. Conflicting results regarding the effects of the same substance on sexual risk behaviors may be due to confounding effects of other substances and the definition of sexual risk behaviors used for that particular study. 

We decided to use the Youth Risk Behavior Surveillance System (YRBSS) database of 2019 to conduct our study. According to the CDC, 'multiple sexual partners' in the YRBSS database are defined as participants with four or more lifetime sexual partners [10]. Our study identified individuals with sexual risk behaviors as participants with multiple sexual partners who did not use a condom during their last intercourse. Using a computed variable that only includes participants with both sexual risk behaviors associated with the highest risk of sexually transmitted infections (STI) and, to a lesser extent, unwanted pregnancy as our cohort, we aim to improve the clinical implications of our results.

## Materials and methods

Details of YRBSS data

The YRBSS contains statistics collected by the CDC to monitor health behaviors that contribute to the leading cause of morbidity and mortality among youth and adults. The six main categories monitored by YRBSS include 1) behaviors that contribute to unintentional injury and violence, 2) tobacco use, 3) alcohol and other drug use, 4) sexual behaviors that contribute to unintended pregnancy and STD/HIV infection, 5) dietary behaviors, and 6) physical inactivity. YRBSS also monitors the prevalence of asthma, obesity, and other health factors. YRBSS uses a three-stage cluster sample design to produce a national representative sample of grade 9-12 students. A new YRBSS database is released every two years. The latest publicly available YRBSS database at the time of this study was the YRBSS 2019 database. The YRBSS data and documentation is available on the CDC website as additional resources, including data documentation and analysis guides.

Study population

We performed a retrospective cross-sectional study using the YRBSS 2019 data, excluding participants with missing information of age, sex, race, grade, and various substance use from the analysis.

Outcomes

The primary aim of this study is to identify the prevalence and characteristic patterns of sexual risk behaviors among US adolescents. The secondary objective is to evaluate the difference in the odds of sexual risk behaviors for various substances.

Sexual risk behaviors

To identify sexual risk behaviors, we used the following two questions from the YRBSS 2019 database: 'Q60. During your life, with how many people have you had sexual intercourse?', 'Q63. The last time you had sexual intercourse, did you or your partner use a condom?'. We identified individuals with sexual risk behaviors as participants who met both the criteria of having four or more lifetime sexual partners and not using condoms during the last intercourse. We believed that including participants with both sexual risk behaviors would increase the generalizability of the results. For this study, we decided to focus on sexual risk behaviors that increase STDs.

Substance use

To identify substance use, we used the following questions from the YRBSS 2019 database: 1) Traditional cigarette: 'Q30. Have you ever tried cigarette smoking, even one or two puffs?', 2) Electronic vapor product: 'Q34. Have you ever used an electronic vapor product?', 3) Marijuana: 'Q45. During your life, how many times have you used marijuana?', 4) Synthetic marijuana: 'Q48. During your life, how many times have you used synthetic marijuana?', 5) Pain medication without prescription: 'Q49. During your life, how many times have you taken prescription pain medicine without a doctor's prescription or differently than how a doctor told you to use it?', 6) Cocaine: 'Q50. During your life, how many times have you used any form of cocaine, including powder, crack, or freebase?', 7) Inhalants: 'Q51. During your life, how many times have you sniffed glue, breathed the contents of aerosol spray cans, or inhaled any paints or sprays to get high?', 8) Heroin: 'Q52. During your life, how many times have you used heroin (also called smack, junk, or China White)?',9) Methamphetamine: 'Q53. During your life, how many times have you used methamphetamines (also called speed, crystal meth, crank, ice, or meth)?', 10) Ecstasy: 'Q54. During your life, how many times have you used ecstasy (also called MDMA)', 11) Anabolic steroids without prescription: 'Q55. During your life, how many times have you taken steroid pills or shots without a doctor's prescription?', 12) Hallucinogens: 'Q91. During your life, how many times have you used hallucinogenic drugs, such as LSD, acid, PCP, angel dust, mescaline, or mushrooms?', 13) Injection drug use: 'Q56. During your life, how many times have you used a needle to inject any illegal drug into your body?', 14) Alcohol: Since there was no direct question regarding alcohol use ever, we used the age of initiation of alcohol to find out participants who ever used alcohol. 'Q40. How old were you when you had your first drink of alcohol other than a few sips?' Responses to all substance-use questions were dichotomized as 'yes' or 'no'.

Covariates and confounders

We included age, sex, race, grade, and concurrent substance use as potential covariates and confounders in our analysis.

Statistical analysis 

We performed all the analysis using IBM SPSS Statistics for Windows, Version 26.0, released 2019 (IBM Corp, Armonk, NY). We used the complex sample addon in SPSS to account for strata, clusters, and sample weight. We derived descriptive statistics using the Rao-Scott chi-square test for categorical variables to determine a statistically significant association. Strata, clusters, and weight accounted for multivariable logistic regression analysis was used to determine the odds of sexual risk behaviors for various substances after adjusting for previously defined covariates and confounders. All statistical tests used were two-sided. P-value <0.05 was considered statistically significant. c-index (area under the receiver operating characteristic (ROC) curve) to evaluate the goodness of fit was calculated for the regression model.

## Results

Epidemiological characteristics of adolescents

Out of 11,919 participants from YRBSS 2019, 463 (3.9%) engaged in sexual risk behaviors. Among the age groups, 17-year-old (37.1% vs. 23.0%; p<0.0001) and >= 18-year-old (26.0% vs. 12.8%; p<0.0001) participants had a higher prevalence of sexual risk behaviors compared to participants who did not meet the criteria for sexual risk behaviors. In addition, the prevalence of sexual risk behaviors was higher in African Americans (16.8% vs. 10.3%; p=0.04). We also found a higher percentage of sexual risk behaviors in participants in grade 11th (27.8% vs. 24.0%; p<0.0001) and grade 12th (47.2% vs. 22.5%; p<0.0001). The epidemiological characteristics of adolescents as per YRBSS 2019 are given in Table 1.

**Table 1:** Epidemiological characteristics of adolescents as per YRBSS 2019 % is column percentage, describing the comparison between sexual risk behaviors and no sexual risk behaviors. Frequency is weighted *p<0.05 **P<0.0001 ^Sexual risk behaviors were defined as participants with four or more lifetime sexual partners who did not use condoms during the last sexual intercourse

Characteristics	Sexual risk behaviors^	No sexual risk behaviors
Weighted frequency (%)	463 (3.9%)	11,456 (96.1%)
Age % **		
<= 14 year old	6.3	12.6
15 year old	7.6	25.8
16 year old	23.1	25.8
17 year old	37.1	23.0
>=18 year old	26.0	12.8
Sex %		
Female	48.2	50.4
Male	51.8	49.6
Race % *		
Black or African American	16.8	10.3
White	48.9	52.6
Hispanic/Latino	7.9	9.3
All other races	26.3	27.8
Grade % **		
9th	8.9	27.5
10th	15.9	25.9
11th	27.8	24.0
12th	47.2	22.5

Prevalence of sexual risk behaviors amongst adolescents with substance use

Prevalence of sexual risk behaviors was higher in participants who used alcohol (92.4% vs. 54.3%; p<0.0001), traditional cigarette (62.2% vs. 21.6%; p<0.0001), e-cigarette (90.3% vs. 48.0%; p<0.0001), marijuana (87.1% vs. 34.7%; p<0.0001), synthetic marijuana (23.7% vs. 6.0%; p<0.0001), pain medication without prescription (36.0% vs. 12.9%; <0.0001), cocaine (27.2% vs. 2.0%; p<0.0001), inhalants (13.7% vs. 5.5%; p<0.0001), heroin (8.7% vs. 0.6%; p<0.0001), methamphetamine (12.6% vs. 0.9%; p<0.0001), ecstasy (22.6% vs. 2.0%; p<0.0001), anabolic steroids without prescription (11.5% vs. 1.1%; p<0.0001), injection drugs (9.7% vs. 0.6%; p<0.0001) and hallucinogens (30.5% vs. 5.5%; p<0.0001). Table 2.

**Table 2:** Prevalence of sexual risk behaviors among adolescents with substance use. % is column %, describing a comparison between sexual risk behaviors and no sexual risk behaviors. Frequency is weighted *p<0.05 **p<0.0001

Substance use	Sexual risk behaviors	No sexual risk behaviors
N (%)	463 (3.9%)	11,456 (96.1%)
Alcohol % **	92.4	54.3
Traditional cigarette % **	62.2	21.6
E-cigarette % **	90.3	48.0
Marijuana % **	87.1	34.7
Synthetic marijuana % **	23.7	6.0
Pain medication % **	36.0	12.9
Cocaine % **	27.2	2.0
Inhalants % **	13.7	5.5
Heroin % **	8.7	0.6
Methamphetamine % **	12.6	0.9
Ecstasy % **	22.6	2.0
Steroids % **	11.5	1.1
Injection drugs % **	9.7	0.6
Hallucinogens % **	30.3	5.5

Multivariable survey logistic regression analysis

After adjusting for age, sex, race, grade and concurrent substance use, odds of sexual risk behaviors were highest in participants who used anabolic steroids (adjusted odds ratio (aOR): 4.87, 95% CI: 2.48-9.57; p<0.0001), followed by cocaine (aOR: 3.80, 1.80-8.00; p=0.001), marijuana (aOR: 3.36, 1.64-6.89; p<0.0001), alcohol (aOR: 2.41, 1.05-5.55; p=0.039), e-cigarette (aOR: 2.05, 1.004-4.19; p=0.049), and traditional cigarette (aOR: 1.58, 1.10-2.28; p=0.016). We did not find statistically significant results for the remaining substances. There was a stepwise increase in odds of sexual risk behaviors in participants aged 16 years (aOR: 4.77, 1.56-14.62; p=0.008), 17 years (aOR: 6.57, 1.94-22.26; p=0.004), and 18 years or older (aOR: 7.36, 2.20-24.67; 0.002). The multivariable survey logistic regression analysis is given in Table 3.

**Table 3:** Regression analysis identifying the difference in odds of sexual risk behaviors for various substances. *p<0.05 **p<0.0001 c-value (area under the receiver operating characteristic (ROC) curve) provides the information regarding the goodness of fit for binary outcome in multiple logistic regression

Parameter	Adjusted odds ratio	95% confidence interval
Model: possibility of involving in sexual risk behaviors=1		
Substance use		
Alcohol *	2.41	1.05-5.55
Traditional cigarette *	1.58	1.10-2.28
E-cigarette *	2.05	1.004-4.19
Marijuana *	3.36	1.64-6.89
Synthetic marijuana	0.84	0.51-1.37
Pain medication	1.15	0.81-1.63
Cocaine *	3.80	1.80-8.00
Inhalants	0.63	0.36-1.09
Heroin	1.42	0.68-2.98
Methamphetamine	1.22	0.41-3.59
Ecstasy	1.40	0.72-2.70
Steroids **	4.87	2.48-9.57
Hallucinogens	1.16	0.83-1.63
Age		
<=14 year	Reference	
15 year	1.23	0.46-3.26
16 year *	4.77	1.56-14.62
17 year *	6.57	1.94-22.26
>=18 year *	7.36	2.20-24.67
Sex		
Female	1.10	0.82-1.46
Male	Reference	
Race		
African American *	2.61	1.46-4.68
White	0.80	0.53-1.21
Latino	0.72	0.40-1.29
Grade		
9th	Reference	
10th	1.02	0.38-2.75
11th	1.02	0.37-2.69
12th	1.47	0.42-5.09
c-value		872

## Discussion

Our study found the prevalence of sexual risk behaviors to be 3.9%. The frequency of sexual risk behaviors was higher in participants aged 17 and 18 or older. In addition, participants with sexual risk behaviors reported a higher frequency of usage of all substances included in our study.

We found several interesting relationships between substance use and sexual risk behaviors, previously defined as having four or more lifetime sexual partners, and those who did not use a condom during their last intercourse. We discovered that non-prescription steroid use was associated with the highest odds of sexual risk behaviors among all substances. Cocaine use had the second-highest odds, while alcohol and e-cigarette use had the third and fourth highest odds of sexual risk behaviors. After adjusting for demographics and other substances, we did not find a statistically significant association between synthetic marijuana, pain medications without prescription, inhalants, heroin, methamphetamine, ecstasy, hallucinogens, and odds of sexual risk behaviors. This result suggests that the effects of synthetic marijuana, pain medications, inhalants, heroin, methamphetamine, ecstasy, and hallucinogens on sexual risk behaviors is confounded by steroids, cocaine, marijuana, alcohol, cigarette, and e-cigarette use.

Our results further the understanding of the association between sexual risk behaviors and substance use. Our analysis also provides novel findings. For example, in our combined model that included all substances, steroid misuse was associated with the highest odds of sexual risk behaviors. Secondly, since we only included participants with the two sexual risk behaviors mentioned earlier as our cohort, our results suggest that steroid use is associated with the highest odds of having four or more lifetime sexual partners and not using condoms during last intercourse. Since the presence of both risky factors can exponentially increase the odds of STDs and unwanted pregnancy (to a lesser extent), our results have better utility in designing preventative policies.

Concerning why steroid misuse confers increased sexual risk behaviors, different potential pathways are studied. Anabolic steroid use is associated with increased impulsivity, verbal aggression, and sexual arousal [11]. Steroid use may induce a hypomanic episode associated with increased pleasure-seeking risk-taking, potentially leading to sexual risk behavior [12]. Although these effects are associated with steroid use, the intensity of these effects varies individually, some having profound psychological effects from steroid use while others have little change from their baseline [13]. It is also possible that we may only observe these effects during the acute phase of steroid use. However, chronic users may not have the same effects [14]. In addition, anabolic steroid users are more likely to use drugs such as marijuana, prescription opioids, cocaine, or heroin [15,16]. Anabolic steroid users may use opioids to counteract insomnia, irritability, depression, and withdrawal from anabolic steroids [17]. Steroid use is also associated with increased odds of using alcohol and other illicit drugs during sex [18]. Therefore, using anabolic steroids seems to be a gateway to other sexual risk behaviors, not just the type of sexual risk behaviors we defined in our study. The other substances that we found to be associated with sexual risk behaviors in this study may have causal paths that could explain the association between substance use and sexual risk behaviors. For example, alcohol intoxication can reduce the drinker's perception of potential risks of engaging in sexual risk behaviors [19]. Cocaine use can increase sexual desire and the probability of engaging in unprotected sex in a dose-dependent manner, primarily when the STD status of the opposite person is not known [20]. Each substance may have its unique effects on the odds of engaging in sexual risk behaviors. It is also possible that engaging in sexual risk behaviors may be associated with subsequent drug use [21]. These youth populations may be alienated from unwanted pregnancy and subsequently attached to other deviant peers facilitating exposure to illicit substances [22]. Other psychosocial factors such as having friends who engage in sexual risk behaviors can increase the odds of engaging in sexual risk behaviors in the presence of heavy substance use [23].

Strengths and limitations

The strength of our study is that it has a large sample size and includes school-going adolescents from all over the US. The large sample size on a national level improves the generalizability of our results. Despite these strengths, our study does have some limitations: First, CDC conducts the YRBSS survey by utilizing high school students. Thus, we could not study the prevalence of substance use in students who do not attend high school. Second, not all states and school districts included all the standard questions on their YRBS questions resulting in missing values for specific variables. Third, the substance abuse questions we utilized in our study were dichotomized. Fourth, our study results are based on a cross-sectional design. Thus, we were not able to establish a temporal relationship between sexual risk behaviors and substance use. Residual confounding is always present.

## Conclusions

Although the prevalence of sexual risk behaviors is low in high school students in the US, the prevalence of substance use is significantly higher in participants who engaged in sexual risk behaviors. Among the different substances, anabolic steroid use has the highest odds of sexual risk behaviors, followed by cocaine, marijuana, alcohol, e-cigarettes, and traditional cigarettes. Therefore, clinicians should remain vigilant for anabolic steroid use when screening adolescents for substance use. We encourage further large-scale randomized studies to study the effects of anabolic steroids on sexual risk behaviors.
